# Diverse Effects on Mitochondrial and Nuclear Functions Elicited by Drugs and Genetic Knockdowns in Bloodstream Stage *Trypanosoma brucei*

**DOI:** 10.1371/journal.pntd.0000678

**Published:** 2010-05-04

**Authors:** Christal Worthen, Bryan C. Jensen, Marilyn Parsons

**Affiliations:** 1 Seattle Biomedical Research Institute, Seattle, Washington, United States of America; 2 Department of Global Health, University of Washington, Seattle, Washington, United States of America; Yale School of Public Health, United States of America

## Abstract

**Background:**

The options for treating the fatal disease human African trypanosomiasis are limited to a few drugs that are toxic or facing increasing resistance. New drugs that kill the causative agents, subspecies of *Trypanosoma brucei*, are therefore urgently needed. Little is known about the cellular mechanisms that lead to death of the pathogenic bloodstream stage.

**Methodology/Principal Findings:**

We therefore conducted the first side by side comparison of the cellular effects of multiple death inducers that target different systems in bloodstream form parasites, including six drugs (pentamidine, prostaglandin D_2_, quercetin, etoposide, camptothecin, and a tetrahydroquinoline) and six RNAi knockdowns that target distinct cellular functions. All compounds tested were static at low concentrations and killed at high concentrations. Dead parasites were rapidly quantified by forward and side scatter during flow cytometry, as confirmed by ethidium homodimer and esterase staining, making these assays convenient for quantitating parasite death. The various treatments yielded different combinations of defects in mitochondrial potential, reactive oxygen species, cell cycle, and genome segregation. No evidence was seen for phosphatidylserine exposure, a marker of apoptosis. Reduction in ATP levels lagged behind decreases in live cell number. Even when the impact on growth was similar at 24 hours, drug-treated cells showed dramatic differences in their ability to further proliferate, demonstrating differences in the reversibility of effects induced by the diverse compounds.

**Conclusions/Significance:**

Parasites showed different phenotypes depending on the treatment, but none of them were clear predictors of whether apparently live cells could go on to proliferate after drugs were removed. We therefore suggest that clonal proliferation assays may be a useful step in selecting anti-trypanosomal compounds for further development. Elucidating the genetic or biochemical events initiated by the compounds with the most profound effects on subsequent proliferation may identify new means to activate death pathways.

## Author Summary

The parasite *Trypanosoma brucei* causes human African trypanosomiasis, which is fatal unless treated. Currently used drugs are toxic, difficult to administer, and often are no longer effective due to drug resistance. The search for new drugs is long and expensive, and determining which compounds are worth pursuing is a key challenge in that process. In this study we sought to determine whether different compounds elicited different responses in the mammalian-infective stage of the parasite. We also examined whether genetic knockdown of parasite molecules led to similar responses. Our results show that, depending on the treatment, the replication of the parasite genomes, proper division of the cell, and mitochondrial function can be affected. Surprisingly, these different responses were not able to predict which compounds affected the long term proliferative potential of *T. brucei*. We found that some of the compounds had irreversible effects on the parasites within one day, so that even cells that appeared healthy could not proliferate. We suggest that determining which compounds set the parasites on a one-way journey to death may provide a means of identifying those that could lead to drugs with high efficacy.

## Introduction

In the absence of a vaccine, control of the lethal disease human African trypanosomiasis (also known as African sleeping sickness) relies on case treatment and elimination of the tsetse fly vector which carries the infecting trypanosomes. However, treatment is hampered by challenges of drug toxicity, resistance, and regimens unrealistic for resource-limited settings. Once the trypanosomes cross the blood brain barrier, the drug melarsoprol is typically used in endemic areas and this treatment alone has approximately 5% mortality [1]. The animal pathogen *Trypanosoma brucei brucei* serves as the common model for the African trypanosomes with only a few differences at the genomic level from the human pathogen *Trypanosoma brucei rhodesiense;* it is slightly less related to the other human pathogen, *Trypanosoma brucei gambiense*. All but one of the currently approved drugs for African trypanosomiasis were discovered via screening for activity against whole cells and their cellular targets remain unknown. For medium and high throughput screening of the pathogenic bloodstream form (BF) of *T. brucei*, recent studies have used ATP levels in the culture as a barometer of parasite growth inhibition [2]. Another assay that has been used relies on the reduction of Alamar blue (resazurin) to a red fluorescent compound in live cells [3], [4]. Neither of these assays specifically assesses parasite death.

Over the past several years, it has become clear that there are many pathways towards death in metazoan cells. Among these are the intrinsic and extrinsic pathways of apoptosis, caspase-independent cell death, oncosis, and autophagy. Potential orthologues of some of the proteins participating in these pathways are encoded in the genomes of African trypanosomes. Examples include the lysosomal cathepsins [5] and endonuclease G [6], which play prominently in caspase-independent cell death. Genes encoding poly(ADP-ribose) polymerase [7] and calpains [8], which play a role in metazoan oncosis, are also present. The set of genes involved in autophagy in *T. brucei* is somewhat reduced [9]; these genes may be involved in life cycle transitions in the parasite [10]. Whether any of these potential mediators play a broad role in the death of BF remains to be seen.

Conversely, genes encoding caspases, which are by definition involved in apoptosis, are absent from the trypanosome genomes. Three metacaspases, which are related to caspases, are expressed in BF, but triple deletion mutants remained sensitive to PGD_2_-induced cell death [11]. Also absent are genes encoding molecules that participate in the extrinsic pathway of apoptosis, such as the death receptors (e.g., Fas receptors) or their downstream mediators, DED domain proteins. The intrinsic pathway of apoptosis involves permeabilization of the outer membrane of the mitochondrion and leakage of components of the intermembrane space into the cytosol. Cytochrome c plays a key role in this pathway, but, unlike procyclic forms, BF lack this protein [12].

In contrast to tens of papers describing programmed cell death (PCD) like phenomena in drug-induced killing of the related parasite *Leishmania*, relatively few studies have explored the pathways of cell death in *T. brucei*. The large majority of these focused on the procyclic stage which differs extensively from the BF stage as to surface molecules and metabolism. Indeed, very few studies have been published concerning pathways of cell death in BF. The phytoflavonol quercetin was reported to kill BF in a mechanism that included phosphatidylserine exposure, a feature commonly seen in mammalian cells undergoing apoptosis [13]. Also of note is a study of prostaglandin D_2_ (PGD_2_) that showed changes in mitochondrial potential, DNA degradation, and phosphatidylserine exposure at the parasite surface [14] in a death process that included oxidative stress [15]. Diamidines, such as pentamidine, were shown to target mitochondrial functions, although they may have additional cellular targets [16]. We were curious as to whether there are distinct pathways of cell death in BF that might be exploited to develop more effective assays for identifying or characterizing potentially useful compounds. We therefore tested a number of potential medium to high throughput assays to see if any would be particularly useful in detecting BF death induced by drugs or RNAi, and if these assays could distinguish different phenotypes of cell death resulting from the various treatments.

## Materials and Methods

### Drugs

PGD_2_ (Sigma) was resuspended in phosphate buffered saline (PBS) to 5 mM. Pentamidine isethionate (Sigma) was resuspended in water to 10 mM. Quercetin dihydrate was freshly dissolved in dimethylsulfoxide to 100 µM for each experiment. Camptothecin (Sigma), etoposide (Sigma) and tetrahydroquinoline compound 4G (kindly provided by Dr. Fred Buckner) [17] were all dissolved in dimethylsulfoxide to 5 mM, 100 mM and 20 mM stocks respectively.

### Strains and media

All work used the BF *T. b. brucei* single marker line, a derivative of the 427 strain that expresses both T7 RNA polymerase and the tetracycline (Tet) repressor, allowing for Tet-regulated expression of transfected sequences [18]. The sole exception is the KREPA3 (systematic ID Tb927.8.620) conditional knockout line, which was constructed in strain 427 [19]. BF, which divide about every eight hours, were maintained at densities below 1.5×10^6^ cells/ml in HMI-9 supplemented with 10% fetal calf serum and 2.5 µg/ml G418. For assays of drug effects, G418 was eliminated from the media. The KREPA3 conditional knockout [19] and PFT-βRNAi (Tb927.7.460) [20] BF lines were gifts of Dr. Kenneth Stuart and Dr. Fred Buckner respectively. The PEX19 (Tb09.211.3300) RNAi BF line was previously described [21]. Cell counts from BF cultures were taken using a Beckman Coulter Counter. To ensure reproducible assays, parasite density was carefully controlled prior to drug treatment or induction of RNAi. Allowing parasites to grow to higher densities in the few days before drug treatment led to higher assay variation. Cultures were diluted to 2×10^5^ cells/ml with fresh media. By 24 hours, the parasite density reached 1−1.4×10^6^ cells/ml. Cultures were again diluted to 2×10^5^ cells/ml and 1 ml of diluted culture per well was placed into 24 well untreated tissue culture plates. Drugs were then added at the indicated concentration (in triplicate) and the parasites were incubated for an additional 24 hours before analysis, except as noted. Alternatively, expression from Tet-regulated constructs was induced with 1–2 µg/ml Tet.

After preliminary studies to determine the approximate plating efficiency of drug-treated cells, limiting dilution analysis as described by Kimball (http://users.rcn.com/jkimball.ma.ultranet/BiologyPages/L/LimitingDilution.html) was conducted by plating three cell concentrations (derived from two separate dilutions series) into 24 wells of a 96-well plate. The proportion of wells without growing cells was determined at day 4 and plotted to allow extrapolation to the plating efficiency. Untreated cells were plated at 0.3, 1 and 1.5 live cells per well for comparison.

### Plasmid construction and generation of RNAi lines

The plasmid pZJM-NOPP44/46 (Tb927.8.760) [22] was transfected into the BF single-marker line as described [23] and modified [24]. Stably integrated plasmids were selected with 2.5 µg/ml phleomycin. For generation of the RNAi constructs to both subunits of TOPIBS (Tb09.160.5070) and TOPIBL (Tb927.4.1330) and the mitochondrial topoisomerase II, TOPIImt (Tb09.160.4090) primers were designed against regions of the genes that had been shown previously to be effective in leading to depletion of the endogenous mRNA when expressed as dsRNA [25], [26]. The forward primers TOPIBS-S (CTCGAGTTATGTGATTTCAGTG), TOPIBL-S (GGTGGCTCATCTTCAGTTG), and TOPIImt-S(TAGGCTTTCAGGGTGAGATACGTC) were used in conjunction with the reverse primers TOPIBS-AS (AAGCTTGTAAACTTCTGGCAGGAC), TOPIBL-AS (AATGGGGACGTTGTTCTCGTTG), and TOPIImt-AS(TTTACGCAAAATGTAGTCGAACGAG) to amplify segments of the genes from genomic DNA isolated from the T. brucei strain 29.13. PCR fragments were directly ligated into *Eam* 1105I digested p2T7^TABlue^-PAC, a derivative of the plasmid p2T7^TABlue^ where the hygromycin resistance gene had been replaced with the puromycin resistance gene (B. Jensen unpublished). Parasites were transfected as described above and transfectants with stably integrated plasmids were selected with 1 µg/ml puromycin. BF transfectants were assumed to be clonal since less than 1/3 of wells yielded transfectants.

### ATP assay

ATP was measured in a bioluminescence 96-well plate assay following the manufacturer's protocol (Roche). All conditions were tested in triplicate. From each triplicate culture, three 25 µl aliquots were flash frozen and stored at −70° until assay. The luminescence obtained was compared with that seen in a dilution series of ATP, as detected by a Fluroskan Ascent Fl luminometer (Thermo Labsystems).

### Flow cytometry

Prior to staining, 10^6^ BF were pelleted by centrifugation for 10 minutesat 3000×g and washed in PSG (phosphate buffered saline with 10 mM glucose) unless otherwise indicated. Samples were analyzed on a Beckman Coulter Epics XL-MCL and analyzed using FlowJo software (Treestar software). Compensation of spectral overlap was performed using single-stained control cells within the FlowJo software. Compensation was manually corrected as needed to ensure that single-stained cells did not fall below the axis. All flow cytometry experiments were performed at least twice for each condition unless otherwise noted.

#### Live/Dead assay

Parasites were resuspended in 0.5 ml PSG. Calcein was added to 0.2 µM (2 µl of 1∶80 dilution of 4 mM stock, Invitrogen) and ethidium homodimer was added to 16 µM (4 µl of 2 mM stock, Invitrogen). Samples were incubated for 20 minutes at room temperature and analyzed immediately by flow cytometry with a 530/40 filter (calcein) and 692/40 filter (ethidium homodimer).

#### Mitochondrial membrane potential

Washed parasites were resuspended in 1 ml PSG, and then mixed with 1 µl of 250 µM rhodamine 123 (Invitrogen) in water. After 20 minutes at room temperature cells were washed with 4 ml PSG, and resuspended in 1 ml PSG for immediate analysis with florescence detected through a 530/40 filter. The relative mitochondrial potential (as compared to untreated cells) was determined, after gating on live cells by forward and side scatter.

#### Phosphatidylserine exposure and protease activation

Washed parasites were resuspended in 0.5 ml annexin binding buffer (10 mM HEPES, 140 mM NaCl, 2.5 mM CaCl_2_, 10 mM glucose. pH 7.4). Parasites were then stained with 4 µl Annexin-V FITC (Invitrogen) and 2 µg/ml propidium iodide (PI, Sigma) for 20 minutes at room temperature. They were then analyzed by flow cytometry with a 530/40 filter (annexin) and 610/20 filter (PI). For analysis of protease activation, 10^6^ treated cells were incubated with 5 µM pan-caspase substrate FITC-conjugated Val-Ala-DL-Asp(O-methyl)-fluoromethylketone (FITC-VAD-fmk) in 0.5 ml PSG for 20 minutes at room temperature. Cells were then washed twice with 4 ml PSG and analyzed by flow cytometry with a 530/40 filter.

#### Reactive oxygen species (ROS)

Washed parasites (2×10^6^) were resuspended with 1 ml PSG and 5-(and-6)-chloromethyl-2′,7′-dichlorodihydrofluorsceine diacetate acetyl ester (CM-H_2_DCFDA) was added to 2 µM. After 10 minutes at 37°, parasites were pelleted and washed with PSG and resuspended in 1 ml HMI-9. After a 30 minute recovery period at 37°, drugs were added. Following drug exposure, cells were again centrifuged, resuspended in 1 ml PSG, and immediately analyzed by flow cytometry with a 530/40 filter.

#### Cell cycle analysis and DNA degradation

Both microscopic and flow analyses were used to assess potential cell cycle defects of the treated parasites. For flow analysis, 10^6^ RNAse-treated parasites were stained with PI as described [27]. The Dean-Jett-Fox algorithm within FloJo was used to estimate proportions of parasites in G1, S and G2/M phases of the cell cycle. For microscopy, parasites were fixed in 2% formaldehyde, washed in 1 ml PBS, and spotted onto multiwell poly-L lysine-coated slides. After 20 min, the liquid was then aspirated and cells were permeablized with 0.1% Triton X-100 in PBS for 5 minutes. After two washes with PBS, samples were stained with 2 µg/ml 4′,6-diamidino-2-phenylindole (DAPI) in PBS for one hour. Slides were then washed twice with PBS, mounted in Prolong antifade reagent (Molecular Probes), and visualized on a Nikon E600 microscope. At least 200 parasites were counted for each condition.

## Results

Our survey of *T. brucei* BF death employed six compounds and six RNAi cell lines, as well as overgrown cultures. For simplicity we refer to these compounds as drugs, although only one, pentamidine is in clinical use for treatment of human African trypanosomiasis (it is used to treat early stage gambiense form human African trypanosomiasis). Of the six drugs examined, three have known mechanisms of action. Tetrahydroquinoline 4G (THQ4G) is an inhibitor of protein farnesyltransferase (PFT) [17] (Fred Buckner, personal communication), which farnesylates specific proteins, facilitating their localization to membranes. The BF drugs camptothecin and etoposide inhibit topoisomerases I and II respectively and camptothecin was previously shown to be toxic to BF trypanosomes [28], [29]. Quercetin and the biological mediator PGD_2_, whose targets are not yet known, were used because of their possible implication in PCD in *T. brucei* [13], [14]. The mode of action of pentamidine is unknown, although it does have mitochondrial targets [16]. Overgrown cultures (post-log phase, densities above ∼2.5×10^6^/ml), which go on to rapidly die via an unknown mechanism, were also examined in some assays.

### Drug treatments and viability

In order to observe processes that occur during BF death, we determined drug concentrations that created a comparable proportion of live and dead cells. We reasoned that such populations would contain some cells in the process of dying. In all drug studies, log-phase BF were used. Parasites were incubated for 24 hours (a normal cell cycle is 6–8 hours) with concentrations of drug spanning EC_50_ values obtained from the literature (see Table 1). At each concentration total number of particles/ml was determined using a Coulter counter. Since this count does not distinguish between live parasites and dead parasites that are relatively intact, a separate live/dead assay was performed to determine the proportion of cell-sized particles that corresponded to live parasites at each drug concentration. The resulting number correlated highly with counts of motile parasites by microscopy using a hemocytometer, but showed less inter-investigator variability.

**Table 1:** Drugs used in this study.

Compound	Target or effect	Published EC_50_	EC_50_ a	EC_75_	Reference
camptothecin	topoisomerase I	1.5 µM	0.55 µM	0.96 µM	[28]
etoposide	topoisomerase II	n.a.	2.6 µM	5.58 µM	n.a.
THQ4G	β subunit of PFT	0.07 µM^b^	0.25 µM	0.45 µM	F. Buckner (personal communication)
quercetin	induces PCD	10 µM	4.8 µM	8.2 µM	[13]
PGD_2_	induces PCD	3.7 µM	3.8 µM	7.4 µM	[14]
pentamidine	unknown	1.7 nM^c^	4.4 nM	7.1 nM	[44]

a As determined by calcein/ethidium assay after 24 hour treatment (see Figure S1).

b The cited study used a 48 hour exposure.

c The cited study used a 72 hour exposure.

n.a., no previous reports were found in literature citing an EC_50_ for etoposide for BF.

**Figure 1:**
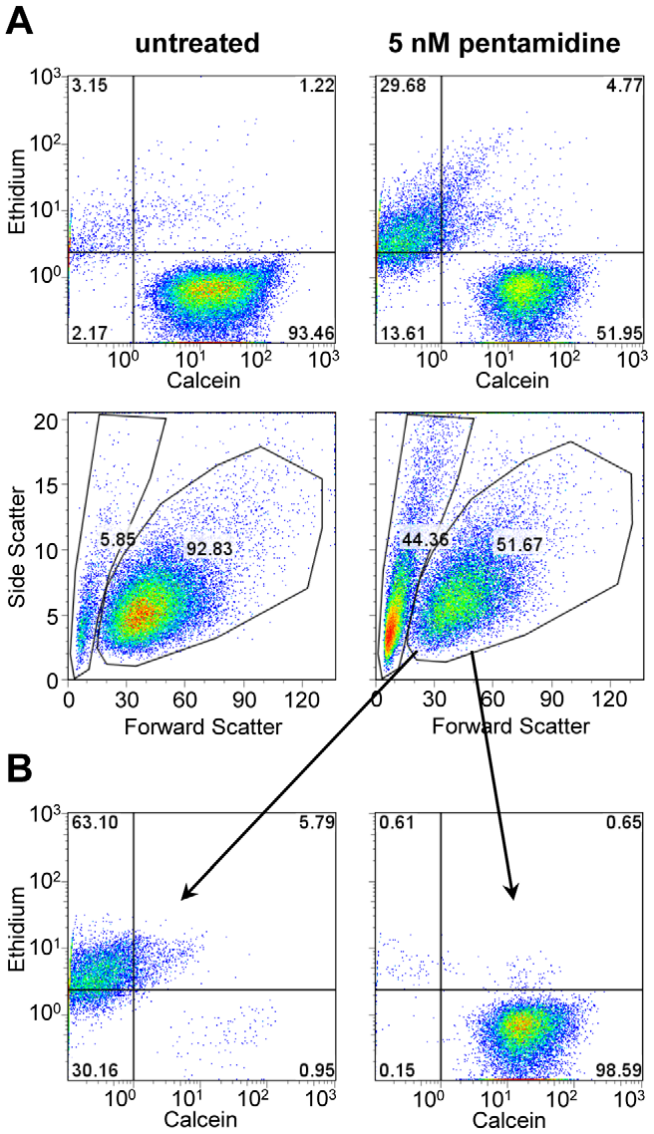
Analysis of BF viability by flow cytometry. A) On the left are untreated cells and on the right are cells treated with pentamidine. The top panels show staining with ethidium (dead cells are permeable to the dye) and calcein (live cells hydrolyze the fluorogenic substrate). The bottom panels show the forward and side scatter, with gates drawn to indicate the region containing live cells. B) The pentamidine-treated cells were divided into two populations by forward/side scatter as shown in Figure 1A and analyzed for calcein/ethidium staining.

The live/dead assay was a calcein/ethidium homodimer double stain. Calcein (a non-fluorescent, cell permeant compound) is cleaved by an esterase in living cells to a green fluorescent compound. Ethidium homodimer is cell-impermeant, and hence its red fluorescence is detected only in dead cells. Although ethidium and related compounds such as propidium iodide are slowly taken up by trypanosomes via facilitated diffusion [30], [31], the background staining in control populations was low compared to the intensity of staining of dead cells (Fig. 1). Most parasites were either calcein-positive, ethidium-negative (alive) or calcein-negative, ethidium-positive (dead). However, some double positive cells were usually seen. We interpret these to be cells that had recently died (were permeable to ethidium), but which had residual esterase activity. In the example of pentamidine treated cultures shown in Figure 1A, there were very few such cells. However, with etoposide, up to half of the ethidium-stained cells showed some, although typically lower, calcein fluorescence (data not shown). Thus, using calcein alone could lead to an overestimate of the proportion of live cells in cultures.

In analyzing the live/dead calcein/ethidium assay, we noted that the live and dead populations of parasites could also be determined by scatter (Figure 1A). By scatter, the parasites grouped into two distinct populations: one with minimal forward scatter, separated by a trough from one showing a broad distribution of forward scatter. The distinction becomes clearer when side scatter is included. Mapping the parasites from the calcein/ethidium assay by scatter demonstrated that 98.8% of the broadly distributed population showing higher forward scatter were calcein-positive (live) parasites (Figure 1B), while less than 1% of the population showing low forward scatter were calcein-positive. This correlation was observed with every drug. Thus we can determine the proportion of live parasites in a population with either the calcein/ethidium assay or by scatter. Using these assays, we were able to assess the number of live cells as compared to the untreated control (Figure S1). Without any drug treatment, the viability of untreated cells was typically greater than 90%. Shown in Table 1 are the determined EC_50_ and EC_75_ for the six drugs along with the published value for each (defined here as a 50% or 75% reduction in number of live cells after a 24 hour treatment). The somewhat higher EC_50_s for THQ4G and pentamidine in the current study likely result from the shorter incubation time used.

For each drug, lower concentrations (<EC_70_) reduced cell numbers without increasing the proportion of dead cells at 24 hours (Figure S2). For all drugs, when the concentration of drugs was increased, the proportion of live cells at 24 h dropped precipitously. To assess loss of viability (as defined by the ability to clonally proliferate), we turned to limiting dilution analysis. Decreasing amounts of drug-treated cultures with known numbers of live parasites were plated into the wells of microtiter plates. The results showed that parasites treated with the different drugs at (or near) the EC_70_ showed quite different plating efficiencies (see Table 2). Camptothecin and quercetin had little impact on cell viability, with most parasites that were alive after exposure able to give rise to colonies. Etoposide and PGD_2_ had a moderate effect on cell viability with approximately 25% of the cells able to recover. THQ4G had a more profound effect on proliferative potential, while pentamidine had a drastic effect. Indeed, we were unable to observe any colonies arising following from pentamidine treatment at EC_75_ implying a plating efficiency of less than 0.05%. Strikingly, even a more mild treatment with pentamidine, which resulted in only a 25% decrease in population growth after 24 hours, still had an extreme effect on the parasites, with only 0.4% of them able to recover and resume growth. Previous studies showed that continued exposure to pentamidine over ten days resulted in a much enhanced effect on *T. gambiense* growth and viability as compared to the effect seen at 24 hours [32]. Our results suggest that this enhancement includes a component resulting from a commitment to a slow death that occurs within the majority of cells in the first 24 hours of pentamidine exposure.

**Table 2:** Plating efficiency following drug treatment.

Compound	Relative inhibition^a^	% dead cells	Plating efficiency (%)
untreated		4.4	95
camptothecin	76.9	29.5	68
etoposide	65.5	23.6	23
THQ4G	69.6	20.8	8
quercetin	69.3	19.8	53
PGD2	69.9	25.7	28
pentamidine	25.5	10.9	0.4
pentamidine	75.8	51.9	<0.02^b^

a Average decrease in number of live cells as compared to untreated cultures, in multiple plating experiments.

b Plating 200 live cells per well (24 wells per experiment) gave rise to no colonies. This number (0.02) assumes the next well would have given rise to a colony.

For the following experiments we aimed for a 75% reduction in the number of “live” cells because that usually gave a good representation of both live and dead cells for the assays. All drug treatments were monitored by cell counting and forward/side scatter to allow comparison of parasite populations showing similar reductions in live cells, unless otherwise noted.

### Effect of drugs on mitochondrial membrane potential, ROS, and ATP levels

**Figure 2:**
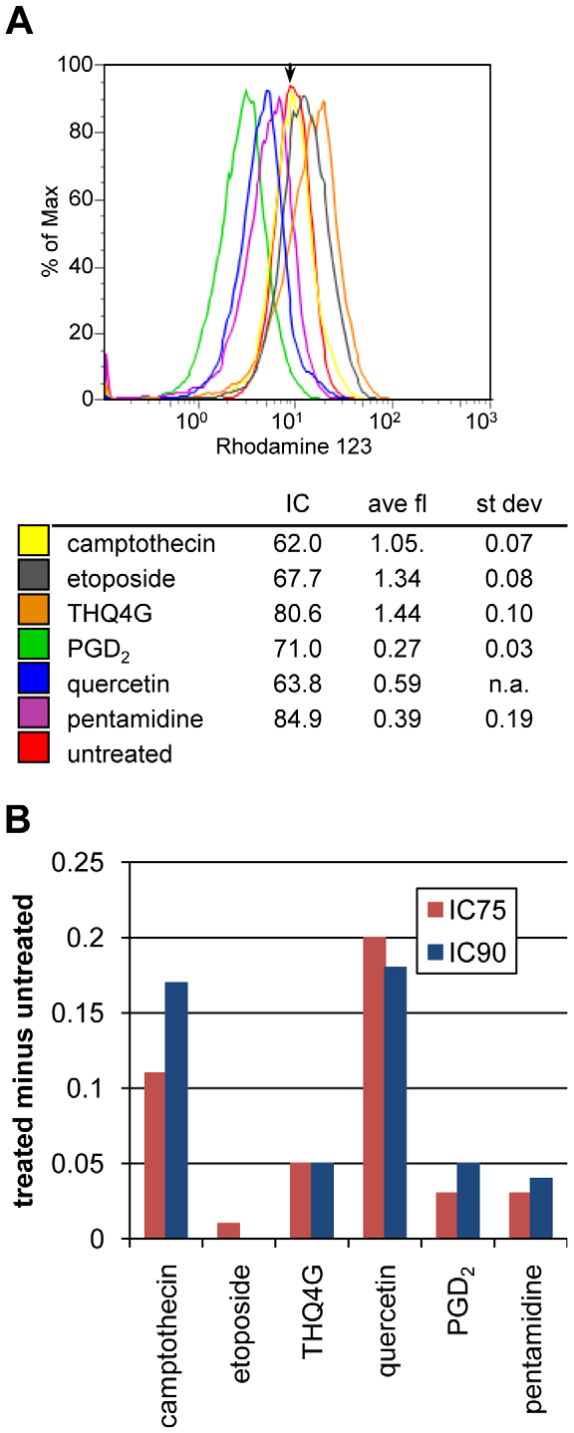
Assays of mitochondrial potential and generation of ROS in drug-treated parasites. A) Mitochondrial potential. After 24 hour drug treatment, parasites were incubated with rhodamine 123 and analyzed by flow cytometry. Dead cells, which were present in all samples, were excluded by gating on scatter. The arrow marks the midpoint for the untreated control. The legend includes the results from multiple experiments, showing the percentage decrease in number of live cells, average and standard deviation of rhodamine 123 fluorescence expressed as the ratio of the geometric means of the treated samples versus the untreated sample. B) Generation of ROS. Amount of ROS above that seen in untreated cells following a two-hour exposure to the indicated drugs at two different concentrations (see Table 1). ROS were assayed by flow cytometric detection of oxidized CM-H2DCFDA.

For a number of drugs that affect viability of trypanosomatids, including pentamidine, the ultimate downstream target has been proposed to be the mitochondrion [16]. As a measure of mitochondrial health and function, we used the cell-permeant fluorescent dye rhodamine 123, which is concentrated in the mitochondria of live cells as a consequence of their mitochondrial membrane potential. Log-phase cells were incubated with each drug for 24 hours and then stained with rhodamine 123. Parasites were analyzed by flow cytometry, gating on live cells as determined by scatter. Shown in Figure 2A is a representative experiment comparing the mitochondrial potential of parasites treated with the various compounds to that of untreated parasites. To allow comparison of experiments done at different times, the geometric mean of each sample was determined and then normalized against an untreated control analyzed at the same time. As seen in Figure 2A, the drugs PGD_2_, quercetin, and pentamidine all induced a loss of mitochondrial membrane potential compared to the untreated control. Camptothecin-treated cells showed no significant difference in potential, while etoposide-treated and THQ4G-treated cells showed slight increases in rhodamine 123 fluorescence. These small increases seem to be the consequence of an increase in cell size as seen by scatter, since when after gating for cells of normal size, the difference disappeared (data not shown).

To assess ROS formation, BF were preloaded with CM-H2DCFDA, which becomes fluorescent upon oxidation. Dye-loaded parasites were then treated for two or four hours with the compounds at concentrations that would yield approximately a 75% or 90% reduction in the number of live cells by 24 hours. In addition, due to the shorter exposure time, the parasites were also tested at twice the 24 hour EC_90_ concentration. As a control, the known oxidizing reagent hydrogen peroxide was applied for one hour (Figure S3 shows the dose-response curve). At the EC_50_, hydrogen peroxide yielded about 3-fold more ROS signal than seen as background in untreated cells. Only two of the compounds, camptothecin and quercetin, showed reproducible fluorescence above background (Figure 2B), and changes were much more modest. However, fluorescence was increased at four versus two hours of treatment, indicating a cumulative effect. In contrast, untreated BF showed decreased fluorescence at four hours, suggesting slow loss of the dye. A previous study [15] showed increased ROS following PGD_2_ treatment, which we did not observe. However, those studies utilized stationary phase BF rather than log phase BF as employed here.

The ATP concentration in aliquots of cells in culture medium was measured after 24 hours of drug treatment as described in the Materials and Methods. When the number of live cells was decreased by approximately 75%, the ATP level also decreased but only about half as much (Table 3). Thus measurement of ATP levels at 24 hours is not as sensitive in revealing drug effects as other methods such as scatter or live/dead staining combined with particle counting. Others have measured ATP levels to assess compound effects in screening assays; those assays used a longer drug exposure period (48 hours) [2] which would magnify the differences between treated and untreated samples. Interestingly, at high parasite density (these cultures had passed out of log phase growth and were at a density of 2.8×10^6^ cells/ml) the ATP level dropped three-fold on a per cell basis, even though the population was still highly viable as measured by forward and side scatter.

**Table 3:** ATP levels in drug-treated cells.

	Relative live cell number	Relative ATP	nM ATP±S.D.
camptothecin	0.22	0.78	10.40±1.42
etoposide	0.28	0.88	11.70±0.63
THQ4G	0.33	0.78	10.42±0.52
quercetin	0.19	0.41	5.46±1.06
PGD_2_	0.57	0.80	10.59±0.96
pentamidine	0.29	0.34	4.57±0.40
untreated	1	1	13.8±2.40

Relative live cell number and ATP are in comparison to untreated cells.

### Effects on cell cycle and organelle segregation

**Figure 3:**
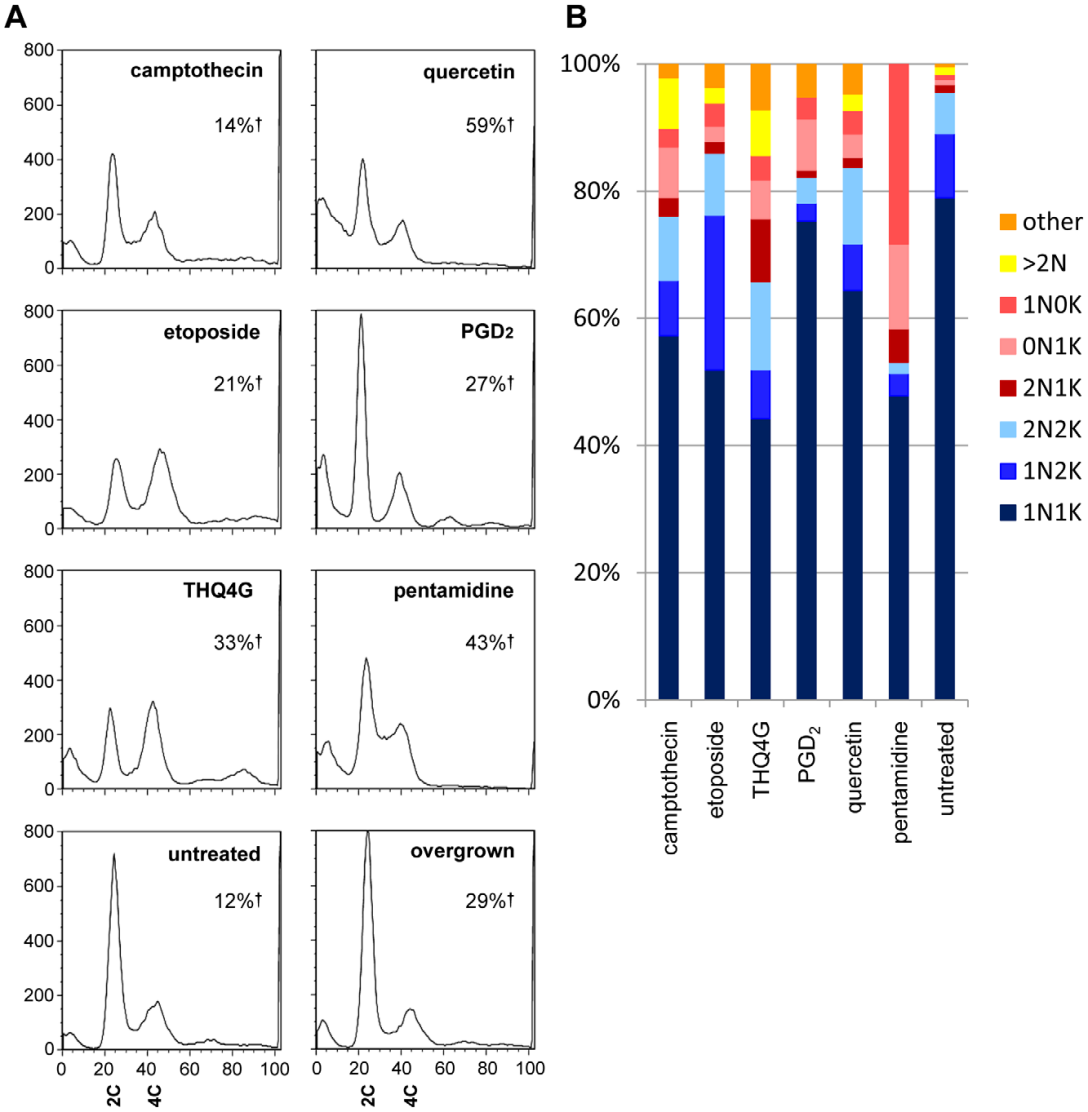
DNA content and genome segregation following drug treatment. A) DNA content following 24 hour treatment revealed by PI staining of RNAse treated BF. The large peak at left seen in some samples represents cells with degraded DNA. 2C indicates diploid (G1) DNA content, 4C indicates G2/M DNA content. Note the appearance of cells with sub-G1 DNA (peaks at far left) following some treatments, as well as cells with higher order DNA content. In each case, the total number of live cells was less than 50% of the untreated controls. The percentage of dead cells (%†) is indicated on each graph. B) Duplication and segregation of the nucleus and kinetoplast following drug treatment. The same populations of cells analyzed in Figure 3A were subjected to microscopic analysis, enumerating the number of nuclei and kinetoplasts per cell as revealed by DAPI staining. Forms seen during normal cell cycle progression are indicated by blue shades, while red to yellow shades indicate abnormal, dead end forms. N, nucleus, K, kinetoplast.

Some drugs affect cell cycle progression or result in degradation of DNA, causing decreased population growth or cell death. Two drugs studied here, the topoisomerase inhibitors etoposide and camptothecin, are known to have such effects in other organisms. *T. brucei* has both a nuclear DNA cycle and a mitochondrial DNA (kinetoplast or kDNA) cycle. The division of the single kinetoplast precedes that of the nucleus, such that parasites progress from a one nucleus and one kinetoplast (1N1K) G1 cell to a 1N2K cell that is in late S-phase or G2, to a 2N2K cell that has completed nuclear division. When the nucleus or kinetoplast fail to replicate, divide, or position properly, aberrant, dead-end progeny result, such as those with two nuclei and one kinetoplast (2N1K) or no nucleus and one kinetoplast (0N1K, also called zoids). Figure 3A shows the DNA content of drug-treated, fixed parasites analyzed by flow cytometry, while Figure 3B shows its cellular distribution visualized by fluorescence microscopy.

As seen in Figure 3A, the untreated populations show a very large G1 peak, a smaller trough containing S phase cells, and a moderately sized G2/M peak. A significant sub-G1 peak is not seen in this healthy population. In contrast, each drug induced a significant sub-G1 peak, which was especially prominent for etoposide, pentamidine, and PGD_2_. This sub-G1 peak would be expected to include dead cells with degraded nuclear DNA as well as anucleate parasites containing only kDNA. Etoposide, and to a lesser extent camptothecin, caused an increase in late S-phase/G2-phase cells, which was expected since they are both topoisomerase inhibitors. Etoposide, which specifically inhibits class II topoisomerases, did not cause a significant increase in abnormal genome segregation patterns at the time point studied but did show a large increase in 1N2K cells, consistent with the G2 arrest seen by flow cytometry. This fits well with the known role of topoisomerase II in untangling chromosomes during mitosis. Pentamidine treatment caused a large increase in the proportion of parasites lacking kDNA as has been previously observed [33], and an increase in the number of cells with S-phase and possibly those with G2/M DNA content. THQ4G also caused a very prominent increase in the G2/M peak and an emergence of a>G2 peak (4N and greater). Microscopic analysis showed that there was a marked increase in parasites with two nuclei and one or two kinetoplasts, correlating to the increased G2/M peak seen on flow cytometry. In addition a number of parasites had more than two nuclei, indicating a defect in cell division. Morphology was dramatically affected by treatment with THQ4G, with many parasites showing a refractile “big eye” appearance that was not seen with the other drugs. The “big eye” phenotype was previously seen in parasites defective in endocytosis; it represents swelling at the flagellar pocket where endocytosis occurs [34]. Quercetin did not have a specific effect on cell cycle as measured by DNA content, while PGD_2_-treated cultures showed a loss of S-phase cells. Overgrown cells showed an increase in G1.

### Assays for PCD

**Figure 4:**
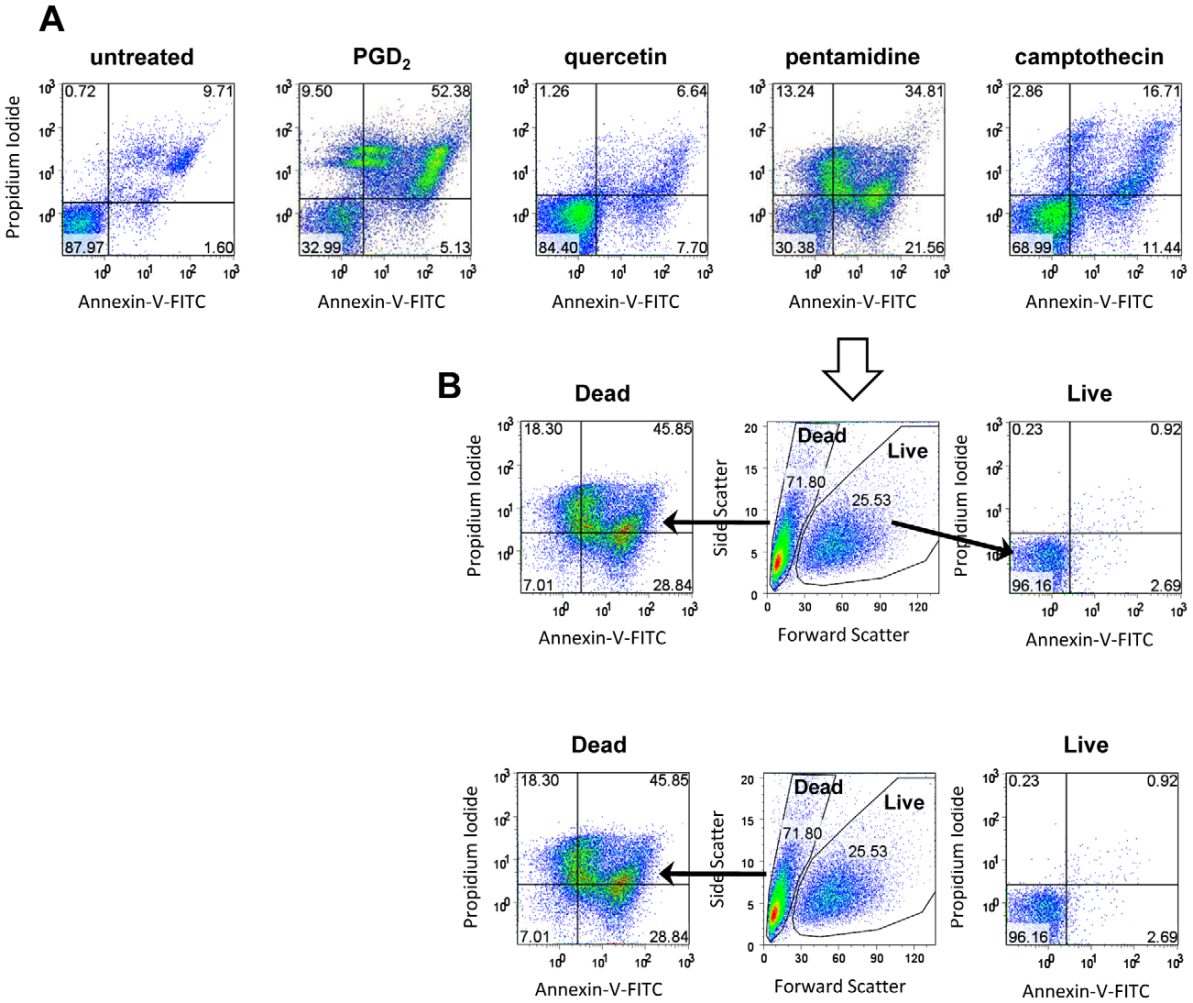
Annexin V staining of populations with dying cells. A) Annexin V and PI staining of unfixed cells. Untreated cells show predominantly annexin V-negative, PI-negative staining (lower left quadrant). All drug treated cells showed additional populations with higher annexin staining coupled with low to high PI staining. B) Annexin-positive cells are dead. The pentamidine-treated population shown in A was analyzed according to light scattering properties. The low forward scatter, dead population (D) contained all annexin-stained cells, including those with low to high PI staining. The live population contained the annexin-negative, PI-negative cells.

Two of the drugs included in these studies, PGD_2_ and quercetin, have been implicated as eliciting a possible PCD mechanism in *T. brucei*. One hallmark of the apoptotic mode of PCD is the depolarization of the plasma membrane, in which the normally cytoplasmically facing phosphatidylserine is flipped to external face of the intact membrane bilayer. Exposed phosphatidylserine can be detected with annexin-V, a cell-impermeant protein. To verify that the cells have not lost membrane integrity (which would allow annexin-V binding to internal phosphatidylserine), the assays used non-fixed cells and included the membrane-impermeable stain PI. A parasite undergoing canonical apoptotic PCD should stain positive for annexin-V, but negative for PI. Under the conditions used, we never observed a clear annexin-positive/PI-negative population. Untreated cultures showed few cells staining with PI or annexin. The treated cultures all had similar patterns of staining (Figure 4A shows results from four drugs). Populations with moderate to high annexin staining emerged, and all showed increased PI staining. When the samples were gated on scatter (Figure 4B), all of the annexin-positive populations showed the low forward scatter indicative of dead cells. The reasons for multiple annexin-positive, PI-positive populations are unclear, but cells with sub-G1 DNA content are clearly present (as seen in Figure 3) and DNA degradation could reduce staining with PI.

A second hallmark of PCD is the activation of specific proteases, most notably caspases. While the *T. brucei* genome does not encode any homologues of classical caspases, it does encode many other proteases including metacaspases. To determine if proteases with caspase-like substrate specificity were activated upon drug treatment, we used the cell permeant pancaspase substrate FITC-VAD-fmk. Cleavage of the fluorogenic substrate leads to increased fluorescence, detectable by flow cytometry. In the related parasite *Leishmania*, it has been observed that treatment of the parasites with nitric oxide, miltefosine, or hydrogen peroxide (albeit at a lower level) can activate a protease that cleaves this substrate (N. Fasel, personal communication). BF cultures treated with hydrogen peroxide had a population of parasites that had significantly increased protease activity directed towards this substrate. However, BF cultures that were treated with the other compounds at their EC_50_ or higher showed no intact cells that had significantly increased fluorescence (data not shown). We did observe that there was a slight increase in fluorescence in the dead cells (those with low forward scatter) regardless of the treatment.

### Depletion of potential drug targets via RNAi

We next used selected assays to examine the consequences of genetic knockdown of genes known or suspected to be essential in BF. The targets of three of the compounds tested above are known: type I topoisomerases, type II topoisomerases, and PFT are the targets of camptothecin, etoposide, and THQ4G respectively. The parasite encodes multiple isoforms of topoisomerases. For the current studies we used RNAi to target topoisomerase IB large and small subunits (TOPIBL and TOP1BS, which gave similar results in all studies) and mitochondrial topoisomerase II (TOPIImt), both of which were previously shown to be essential in procyclic forms [25], [26], [35]. We also targeted the beta subunit of PFT.

**Figure 5:**
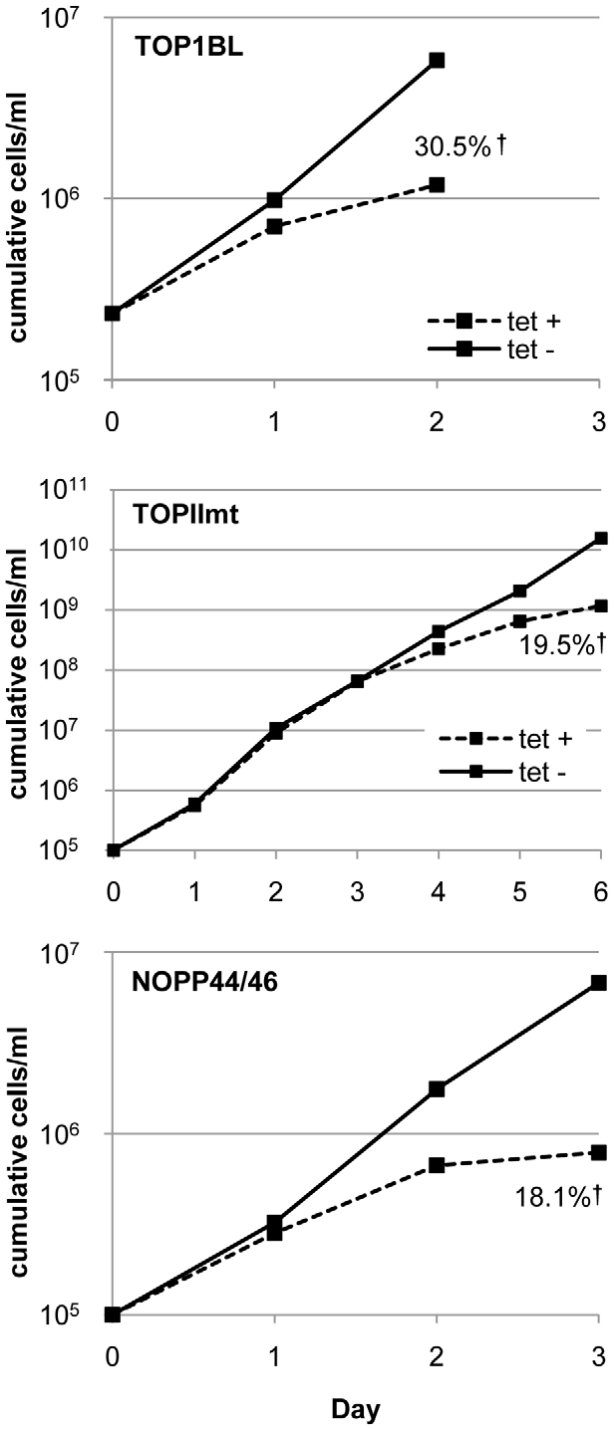
Representative growth curves for RNAi cell lines targeting TOPIBL, NOPP44/46, or TOPIImt. Tet was added at day 0 to initiate destruction of the targeted RNA. The RNAi-induced cells from the final day of the growth curve were used in the experiments shown in Figure 6. The percent of dead cells at that point is listed. NOPP44/46 protein levels were reduced 44% as shown by immunoblotting (unpublished results).

Three other RNAi or conditional knockout lines were chosen for analysis because they target disparate key functions of the cell. PEX19 is involved in targeting of proteins to the glycosome, which compartmentalizes the first steps of glycolysis and glycerol metabolism [21], [36]. Failure to properly localize glycosomal proteins leads to the accumulation of toxic levels of intermediates in these pathways [37] and cell death [38]. KREPA3 is a component of the mitochondrial RNA editosome, which prepares mitochondrial transcripts for translation by insertion or deletion of uridines [19]. The nucleolar protein NOPP44/46 is involved in biogenesis of the large ribosomal subunit [22]. For RNAi lines, addition of Tet to the media induces knockdown of the corresponding mRNA. For the conditional knockout of *KREPA3*, removal of Tet blocks the expression of the complementing gene. Although some of these genes had not previously been tested for essentiality in BF (*TOPIBL* and *TOP1BS*, *TOPIImt*, and *NOPP44/46*), all strains showed compromised growth after disruption of gene expression (Figure 5 and Figure S4). From initial growth curves, we determined when the growth of the strains was strongly affected, which varied depending on the gene. Within the period when population expansion was strongly impacted and before resistant populations emerged, samples were analyzed with selected assays.

In most cases, if knockdown dramatically reduced the numbers of parasites, then it was also accompanied by cell death. A notable exception was the knockdown of PFTβ, which showed little cell death even though cell numbers were decreased by over 60% by day 3. The amount of cell death varied among the other RNAi cell lines, as assessed by either forward/side scatter or calcein/ethidium (the two methods agreed closely). The proportion of live parasites by these two approaches also agreed closely with the proportion of annexin-negative, PI-negative parasites. Similar to what was seen following treatment with drugs, there was not clear evidence for exposure of phosphatidylserine on intact parasites (data not shown). The well-known propensity of *T. brucei* to become resistant to the effects of specific RNAi would make limiting dilution analysis difficult to interpret, so such studies were not conducted.

### Effect of RNAi on mitochondrial potential

As seen in Table 4, depletion of either subunit of TOPIB led to a slight increase in mitochondrial membrane potential, which corresponded to a small increase in cell size. This result is consistent with what was seen for cells treated with camptothecin. In contrast, cells depleted for either TOPIImt, and to a lesser extent, PFTβ, lost mitochondrial potential, a feature that was not evident when cells were treated with drugs that inhibit these enzymes. Down-regulation of the three other targets, KREPA3, PEX19 and NOPP44/46, also resulted in at least a two-fold loss of rhodamine 123 fluorescence.

**Table 4:** Effect of depletion of target molecules on parasite growth, viability, mitochondrial potential.

Gene	Relative live cell number^a^	% dead cells	Relative mito ψ^b^
*TOPIBS*	n.d.	35	1.10
	0.16	28	1.34
*TOPIBL*	n.d.	72	1.33
	0.21	28	0.93
*TOPIImt*	0.08	44	0.44
	0.51	19	0.33
*PFTB*	0.36	14	0.72
	0.3	17	0.68
*PEX19*	n.d.	64	0.47
	0.26	61	0.52
*KREPA3*	0.05	37	0.19
	0.15	19	0.41
*NOPP44/46*	0.04	26	0.34
	0.12	17	0.53

a Defined as the cumulative reduction in live cell number relative to uninduced controls at day of assay. *TOP1BS*, *TOP1BL*, and *PEX19* knockdowns were assayed on day 2 post induction of RNAi, while *PFTB* and *NOPP44/46* knockdowns were assayed on day 3 and *TOPIImt* on day 6. *KREPA3* was assayed five days after removal of Tet.

b as compared to control cells in the same experiment.

### Effect of target down-regulation on cell cycle and genome segregation

**Figure 6:**
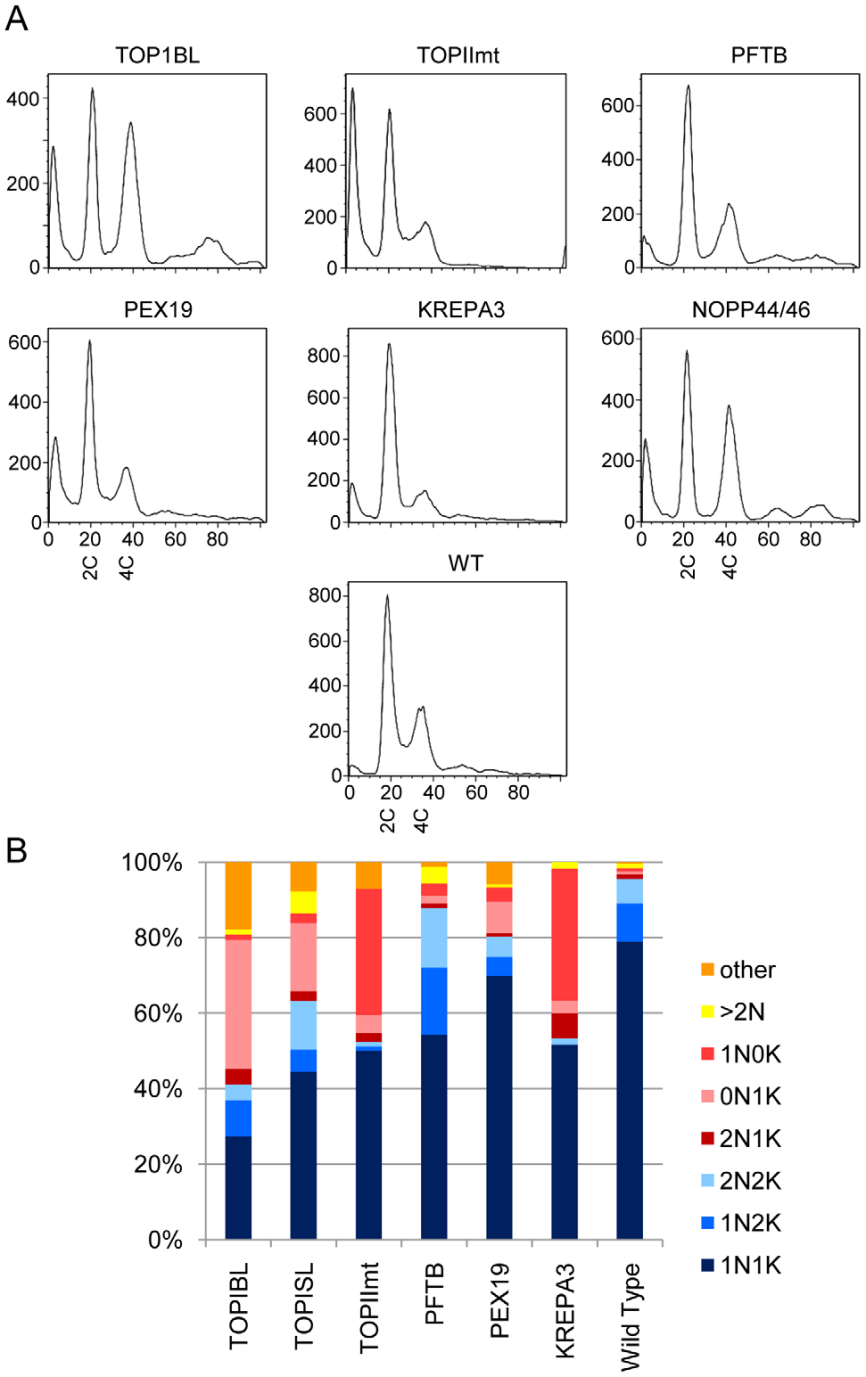
DNA content and genome segregation following genetic knockdown. The same parasites described in Table 4 were analyzed for DNA content (Panel A) and nuclear and kinetoplast genome segregation (Panel B) as described in Figure 3. The cumulative number of live cells was between 10% and 50% of control uninduced populations on the day assayed.

Down-regulation of most of the targets led to a large increase in the sub-G1 peak (Figure 6A), indicating DNA degradation or production of anucleate parasites, as well as defects in organellar replication or segregation (Figure 6B). Induction of RNAi for the large subunit of TOPIBL (and TOP1BS, data not shown) yielded the most aberrant profiles. These populations had the largest sub-G1 peak, plus a dramatic increase in the number of parasites lacking nuclear DNA (0N1K). They also showed an increased proportion of cells with 4C (G2/M) or 8C DNA content in the absence a corresponding increase in the number of nuclei, indicating a mitotic defect. The NOPP44/46 knockdown also showed an enrichment of G2/M phase cells coupled with fewer G1 and S phase cells. In contrast, decreased KREPA3 or TOPIImt resulted in a fewer cells in G2/M phase. These mutants also showed a massive increase in cells lacking a kinetoplast (0N1K). PFT-β RNAi resulted in decreased S phase combined with an increase in 1N2K and 2N2K cells. However, there was no major increase in aberrant segregation events, except for a higher level of multinucleate cells. PEX19 RNAi lines did not show any major changes in the cell cycle.

## Discussion

In this report we examined the effect of six drugs and six genetic knockdowns on multiple cellular parameters in an effort to define characteristics accompanying death of *T. brucei* BF, particularly those that might be amenable to assessment in medium- to high-throughput screens. All of the drugs were apparently static at lower concentrations, but killed at higher concentrations. ATP levels were a less sensitive measure of the numbers of live parasites than the combination of particle counting and flow analysis. Several explanations could be posed for the lag in ATP reduction as measured, such as enlarged cell size (as with etoposide and camptothecin), release of ATP by dying cells, decreased consumption of ATP (due to cessation of energy intensive processes) or, perhaps less likely, increased production of ATP. We found that flow assays detecting decreased forward scatter, increased ethidium or PI staining, and increased annexin V staining all identified dead BF effectively. Coupling forward and side scatter or ethidium with calcein staining provides a clear two dimensional separation of the live and dead populations by flow cytometry. Importantly, when tested at similar ECs, the plating efficiencies of apparently live parasites varied widely depending on the compound used, indicating that the relative number of live cells does not necessarily predict the long term effects of a brief drug treatment. Similarly, none of the other assays (ATP levels, ROS, cell cycle, or genome segregation) were able to clearly predict which of the compounds in this survey would have strong irreversible effects. Thus, clonal proliferation assays may be a useful step in down-selection of compounds identified by high-throughput screening.

The diverse cellular effects following the different treatments are summarized in Table 5. The six drugs fell into two broad groups; those that affected mitochondrial membrane potential, and those that decreased the G1/G2 ratio. The three drugs of unknown mode of action (PGD_2_, quercetin, and pentamidine) behaved similarly. These drugs caused a loss of mitochondrial membrane potential and marked DNA degradation, but had relatively little effect on the G1/G2 ratio or cellular morphology prior to death. In comparison, the drugs THQ4G, etoposide, and camptothecin all affected cell morphology and lead to an increase in the relative numbers of G2 cells. For example, etoposide and camptothecin both created larger cells and G2 arrest, with etoposide doing so to a greater extent. THQ4G produced the largest effect on morphology, with large round “BigEye” cells, huge G2 peaks and a prominent >2N peak detected by flow cytometry. One compound from each group caused modestly increased ROS: quercetin and camptothecin. One compound from each group led to highly decreased viability: pentamidine and THQ4G. Thus a variety of phenotypes were apparent when the parasites were treated with the toxic compounds even though the decrease in the numbers of live cells was similar at 24 hours.

**Table 5:** Summary of differential effects of drugs and RNAi on *T. brucei* BF.^a^

Drug/RNAi	target	Plating efficiency	Mito potential	ROS	Cell cycle	Nucleus+kDNA
campothecin	Top I	−	−	+	+	+
etoposide	Top II	+	−	−	+++	+
THQ4G	PFT	+++	−	−	+++	+++
pentamidine	?	+++	+++	−	+++	+++
PGD_2_	?	+	+++	−	+++	+
quercetin	?	−	+	+	−	+
Reg. knockout	KREPA3	nd	+++	−	+++	+++
RNAi	TOPIImt	nd	+++	−	+++	+++
RNAi	TOPIB	nd	−	nd	+++	+++
RNAi	PFT-β	nd	+	nd	+	+
RNAi	NOPP44/46	nd	+++	−	+++	nd
RNAi	PEX19	nd	+++	−	−	+

a –, no effect; + limited effect; +++, significant effect following RNAi or 24 hour treatment with drugs at IC_75_ concentration were defined as noted below. Not done, nd.

- plating efficiency: percentage of cells categorized as live by scatter able to form colonies, compared to untreated. Limited effect, 2–5 fold reduction; significant effect, >5-fold reduction.

- mitochondrial potential: limited effect, 30−50% reduction; significant effect, >50% reduction.

- ROS: limited effect, 5−30% increase in CM-H_2_DCFDA staining; significant effect, greater than 30% increase.

- cell cycle profile among cells with G1 content or greater: limited effect, G1/G2 or S/G1+G2 1.5−2 standard deviations from untreated cells; significant effect, >2 standard deviations from untreated cells.

- Nucleus+kDNA: limited effect, 10−30% cells having abnormal numbers of nuclei or kinetoplasts, significant effect >30% abnormal forms.

The results with topoisomerases and farnesyl transferase highlight some challenges in predicting drug inhibition phenotypes from RNAi and vice versa. The only drug-RNAi comparison which is predicted to be unambiguous is THQ4G and PFT-β. However, the PFT-β RNAi line did not show the dramatic morphological effect that THQ4G did, and despite strongly decreased growth, the knockdown did not evoke a large amount of cell death. Many explanations can be proposed for this finding, such as sufficient residual PFT-β after RNAi to allow viability (similar effects might be seen at low drug concentrations). Additionally, rapid inhibition via drugs may not allow compensatory changes that could occur during slower loss of activity as in RNAi knockdowns. Alternatively, the stronger effect of the drug could result from off-target effects or the cellular consequences of an inactive, drug-bound protein as opposed to the absence of the protein.

Although the effectiveness of TOPIImt RNAi was clearly evident in the strongly reduced mitochondrial membrane potential and production of anucleate cells, it had a less pronounced effect on BF proliferation than did the topoisomerase II inhibitor etoposide. This suggests that the primary target of etoposide in BFs is not TOPIImt, but rather the nuclear topoisomerase IIα, which was previously shown to be essential in procyclic forms [29]. Conversely, induction of RNAi against TOPIB large or small subunits led to more dramatic cell cycle perturbations than did camptothecin. This was unanticipated since, in addition to topoisomerase IB [25], camptothecin is expected to act on all three type IA/III enzymes, including an essential mitochondrial isoform [39]. Indeed camptothecin form adducts with both kDNA and nuclear DNA in BF [28], indicating action against nuclear and mitochondrial isoforms.

Our study, which examines some of the commonly used assays, shows that different treatments elicited somewhat different response profiles in BF. The large number of differences between BF and procyclic forms in metabolism and surface molecules, as well as host environment, could mean that mechanisms of parasite death vary between the two stages in this species as well. Still it is possible that cellular events precipitated by drugs or genetic intrusions ultimately rely on limited number of pathways to cause cell death. Clearly, there are many other parameters that could be examined in efforts to elucidate such pathways in the future. Whether these responses result from the induction of genetic programs as opposed to biochemical cascades remains to be studied.

Loss of membrane asymmetry, with the exposure of phosphatidylserine on the outer leaflet of the plasma membrane, is considered to be a hallmark of apoptotic cell death. The externalization of phosphatidylserine is thought to involve activation of lipid scramblases [40] (apparently lacking in *T. brucei*) and/or inactivation of aminophospholipid translocases [41] (several of which appear to be present in *T. brucei*). Our studies did not reveal any evidence for exposure of phosphatidylserine except when the plasma membrane was compromised. These conditions included treatment with PGD_2_ and quercetin, both of which have been implicated in a possible PCD pathway in *T. brucei* BF. Although work by previous authors showed evidence of phosphatidylserine exposure upon treatment with these drugs, those studies used stationary phase BF whereas we employed log phase parasites. Because stationary phase BF go on to die relatively rapidly even in the absence of drugs, their death pathways might be somewhat different to what is seen in log phase. More recently, evidence has been presented for phosphatidylserine exposure in the absence of significant membrane permeabilization in BF treated with cordycepin [42] or subjected to endoplasmic reticulum stress [43]. This finding suggests that under certain situations loss of membrane asymmetry may occur as BF die. None of the compounds we studied evoked protease activity against the pan-caspase substrate FITC-VAD-fmk. While we were unable to find evidence for a classical apoptotic pathway, some drugs did elicit a set of apparently live cells that were unable to further proliferate, suggesting that a genetic or biochemical program had been initiated during the initial insult. Whether such programs directly parallel those seen in metazoa is open to question.

All of the compounds we employed killed cells under acute treatment, but only pentamidine is used for treatment of human African trypanosomiasis. Following exposure to this drug, and to a lesser extent THQ4G, cells that retained all of the markers of viability had nevertheless started down a one-way journey to their demise. Further knowledge of how to precipitate the events initiated by these compounds would provide important clues for designing new anti-trypanosomal drugs. The strategy of antigenic variation employed by the parasites means that little help is available from adaptive immunity, raising the bar even higher for drug efficacy.

## Supporting Information

Figure S1 Titration curves for drugs. The effects of drugs were monitored by flow cytometry to detect live and dead cells.
(0.02 MB PDF)

Figure S2 The proportion of live cells compared to inhibition of population expansion. The effects of different concentrations of drugs on the total number of live parasites and the proportion of live versus dead cells in the population.
(0.03 MB PDF)

Figure S3 Titration of hydrogen peroxide in the ROS assay. The effect of hydrogen peroxide in ROS and live/dead assay is shown.
(0.06 MB PDF)

Figure S4 Growth curves for genetic knockdowns. Growth curves for cell lines not depicted in Fig. 5. The cells from the final day of the growth curve were used in the experiments shown in Figure 6.
(0.04 MB PDF)
