# Supplementary material for: Diverse Effects on Mitochondrial and Nuclear Functions Elicited by Drugs and Genetic Knockdowns in Bloodstream Stage Trypanosoma brucei
Source: PLoS Negl Trop Dis. 2010 May 4;4(5):e678. doi: 10.1371/journal.pntd.0000678 (PMC2864271; doi:10.1371/journal.pntd.0000678)
Supplement: Figure S1 — Titration curves for drugs. The effects of drugs were monitored by flow cytometry to detect live and dead cells. (0.02 MB PDF) [file pntd.0000678.s001.pdf]

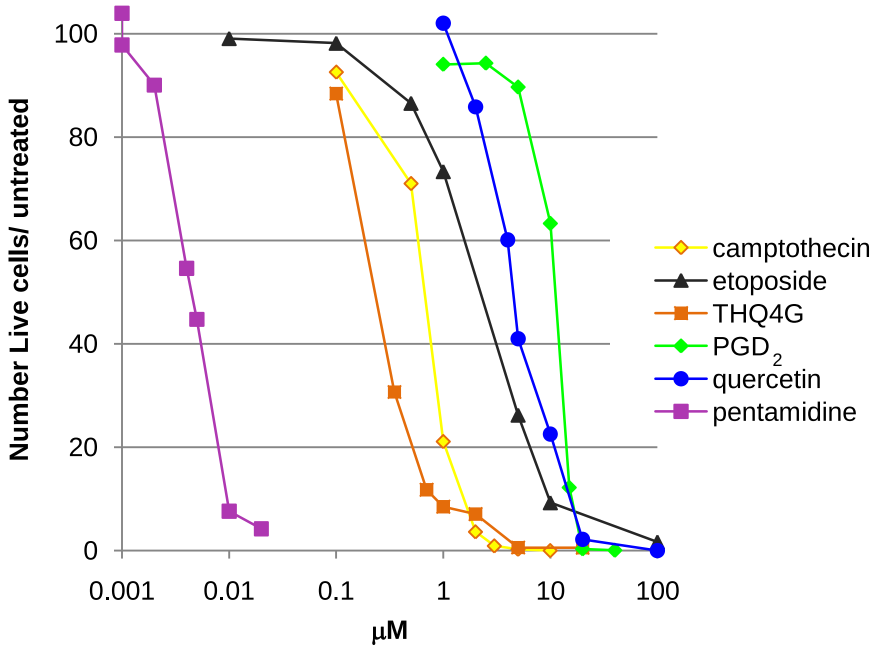

Fig. S1. Titration curves for drugs. Triplicate cultures of BF were analyzed following 24 hour treatments with the indicated concentrations of drugs. The Y axis represents the number of live cells compared to the untreated control. The number of live cells was determined by a combination of Coulter counting and the percent live cells as determined by calcein/ ethidium homodimer staining as revealed by flow cytometry as described in Methods.
