# Supplementary material for: Diverse Effects on Mitochondrial and Nuclear Functions Elicited by Drugs and Genetic Knockdowns in Bloodstream Stage Trypanosoma brucei
Source: PLoS Negl Trop Dis. 2010 May 4;4(5):e678. doi: 10.1371/journal.pntd.0000678 (PMC2864271; doi:10.1371/journal.pntd.0000678)
Supplement: Figure S2 — The proportion of live cells compared to inhibition of population expansion. The effects of different concentrations of drugs on the total number of live parasites and the proportion of live versus dead cells in the population. (0.03 MB PDF) [file pntd.0000678.s002.pdf]

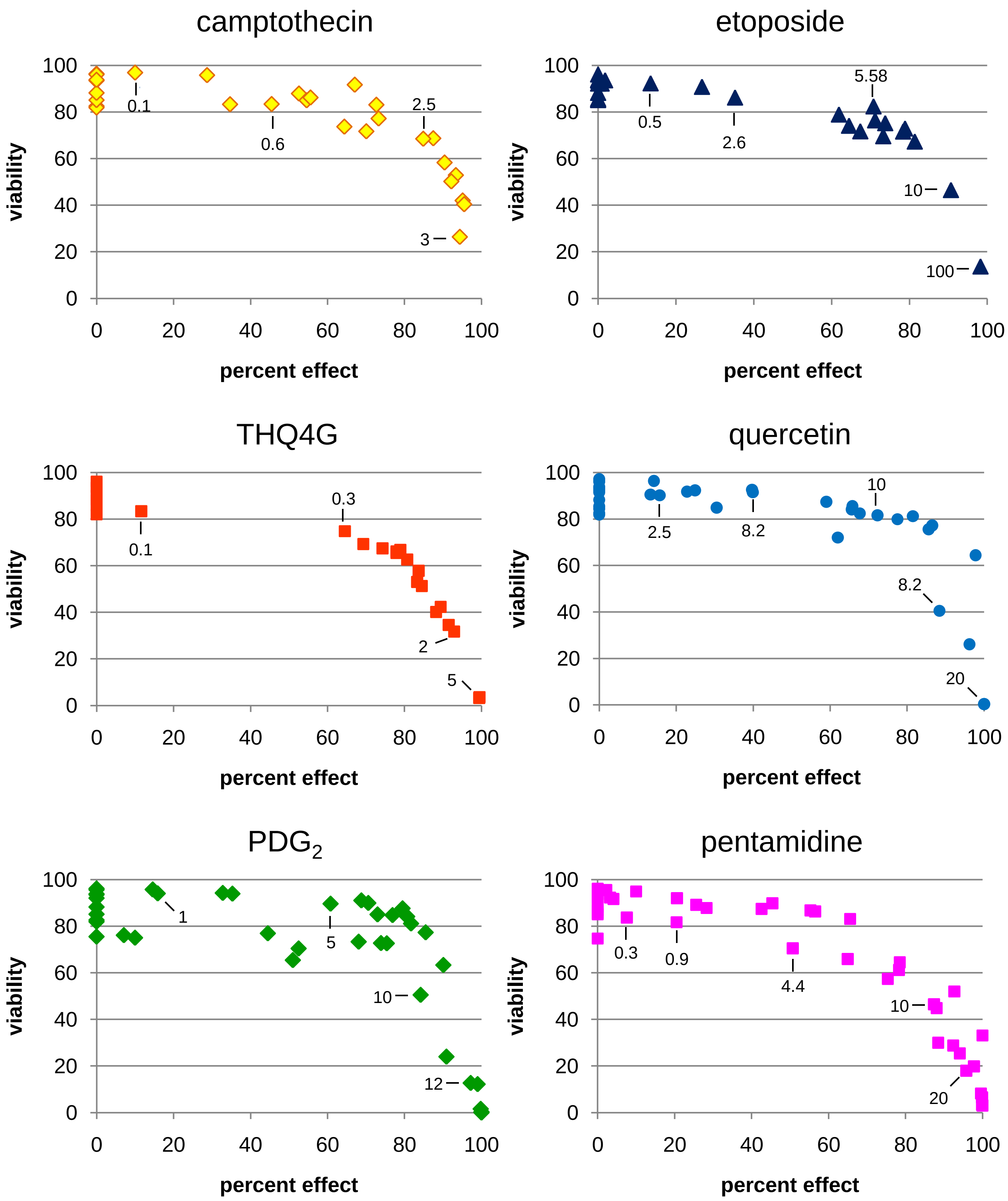


**Fig. S2. Proportion of live cells compared to inhibition of population expansion**. The proportion of live cells following 24 hour drug treatment was determined by forward/side scatter or calcein/ethidium live/dead assay by flow cytometry (Y-axis). The number of live cells was determined by multiplying the total cell count (obtained by Coulter counting) by the fraction that is alive. This number was expressed as a percentage decrease in the total number of live cells as compared to the untreated sample (X-axis). Drug concentrations (in $\mu$M, except pentamidine in nM) of selected points are indicated. The data were accumulated from analyses conducted over many experiments, with each point representing the mean of triplicates in a specific day.
