# Supplementary material for: Diverse Effects on Mitochondrial and Nuclear Functions Elicited by Drugs and Genetic Knockdowns in Bloodstream Stage Trypanosoma brucei
Source: PLoS Negl Trop Dis. 2010 May 4;4(5):e678. doi: 10.1371/journal.pntd.0000678 (PMC2864271; doi:10.1371/journal.pntd.0000678)
Supplement: Figure S3 — Titration of hydrogen peroxide in the ROS assay. The effect of hydrogen peroxide in ROS and live/dead assay is shown. (0.06 MB PDF) [file pntd.0000678.s003.pdf]

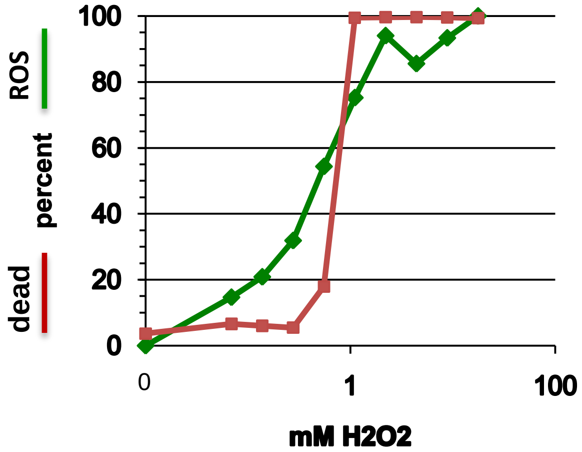

Fig. S3. Titration of hydrogen peroxide in ROS assay. The relative fluorescence yielded by oxidation of H2DCFDA was measured by flow cytometry after one hour (ROS). Percentages were based on untreated cells and cell treated with highest concentration of H_2O_2 (18mM), which yielded the maximum signal. The proportion of dead cells was determined by forward and side scatter.
