# Supplementary material for: Diverse Effects on Mitochondrial and Nuclear Functions Elicited by Drugs and Genetic Knockdowns in Bloodstream Stage Trypanosoma brucei
Source: PLoS Negl Trop Dis. 2010 May 4;4(5):e678. doi: 10.1371/journal.pntd.0000678 (PMC2864271; doi:10.1371/journal.pntd.0000678)
Supplement: Figure S4 — Growth curves for genetic knockdowns. Growth curves for cell lines not depicted in Fig. 5. The cells from the final day of the growth curve were used in the experiments shown in Figure 6. (0.04 MB PDF) [file pntd.0000678.s004.pdf]

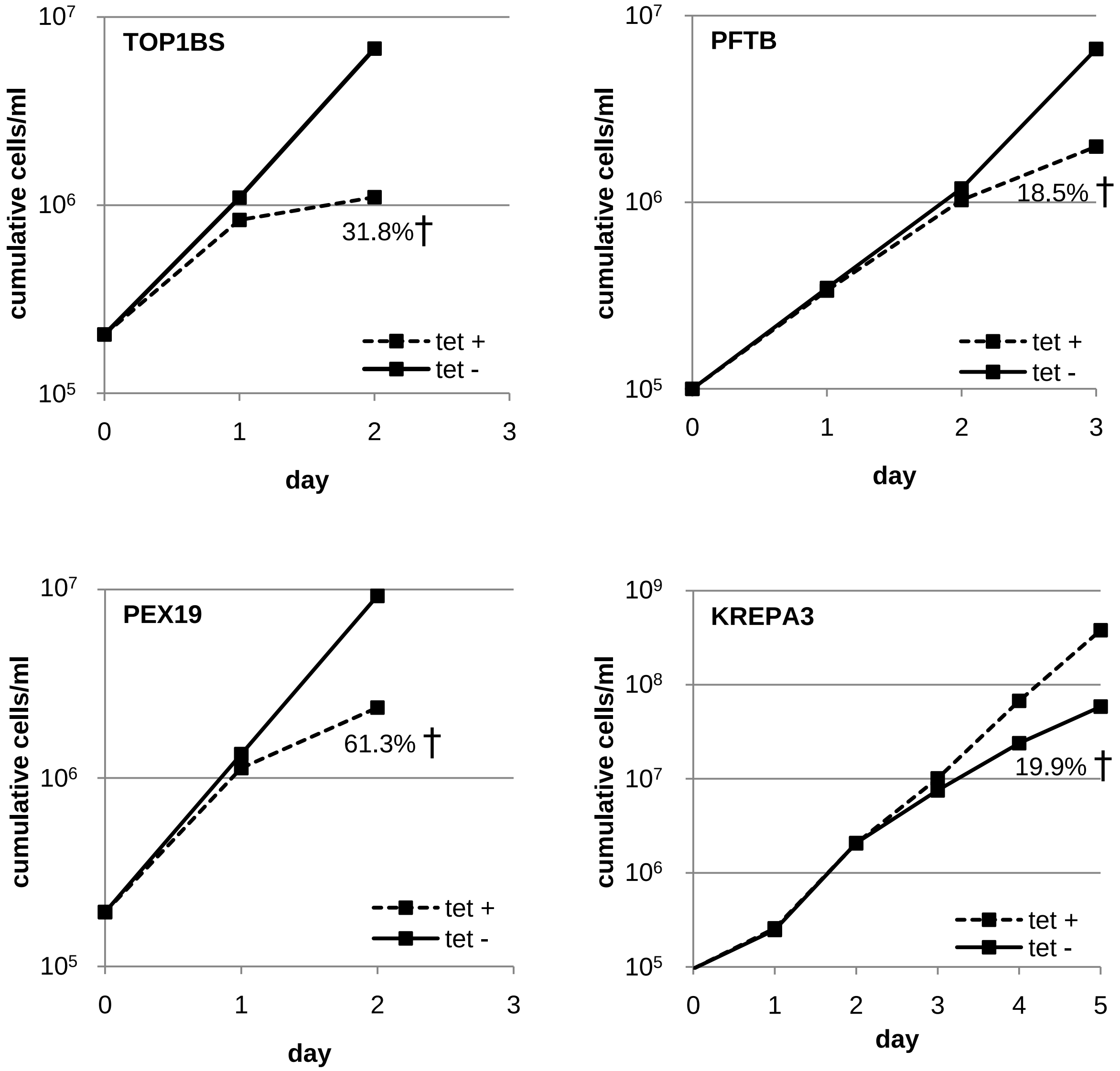

Fig. S4. Growth curves for remaining genetic knockdowns. All cell lines were Tet-induced RNAi knockdowns, except KREPA3, in which Tet induced expression of KREPA3. The total number of cells was assessed by particle counting and includes both live and dead cells. The percent of dead cells (as determined by forward/side scatter) in the non-permissive condition at day of assay is listed next to the cross symbol. The cells from the final day for which data is shown were then used in the experiments shown in Figure 6. Previous studies using these cell lines showed that the abundance of mRNAs encoding PFTβ and PEX19 were reduced 74% [20] and 70% [21] respectively and that the KREPA3 protein was not detectable [19].
